# PALADIN: protein alignment for functional profiling whole metagenome shotgun data

**DOI:** 10.1093/bioinformatics/btx021

**Published:** 2017-01-31

**Authors:** Anthony Westbrook, Jordan Ramsdell, Taruna Schuelke, Louisa Normington, R Daniel Bergeron, W Kelley Thomas, Matthew D MacManes

**Affiliations:** 1 Department of Computer Science, University of New Hampshire, Durham, NH, USA; 2 Hubbard Center for Genome Studies, University of New Hampshire, Durham, NH, USA; 3 Department of Molecular Cellular and Biomedical Sciences, University of New Hampshire, Durham, NH, USA

## Abstract

**Motivation:**

Whole metagenome shotgun sequencing is a powerful approach for assaying the functional potential of microbial communities. We currently lack tools that efficiently and accurately align DNA reads against protein references, the technique necessary for constructing a functional profile. Here, we present PALADIN—a novel modification of the Burrows-Wheeler Aligner that provides accurate alignment, robust reporting capabilities and orders-of-magnitude improved efficiency by directly mapping in protein space.

**Results:**

We compared the accuracy and efficiency of PALADIN against existing tools that employ nucleotide or protein alignment algorithms. Using simulated reads, PALADIN consistently outperformed the popular DNA read mappers BWA and NovoAlign in detected proteins, percentage of reads mapped and ontological similarity. We also compared PALADIN against four existing protein alignment tools: BLASTX, RAPSearch2, DIAMOND and Lambda, using empirically obtained reads. PALADIN yielded results seven times faster than the best performing alternative, DIAMOND and nearly 8000 times faster than BLASTX. PALADIN's accuracy was comparable to all tested solutions.

**Availability and Implementation:**

PALADIN was implemented in C, and its source code and documentation are available at https://github.com/twestbrookunh/paladin

**Supplementary information:**

Supplementary data are available at *Bioinformatics* online.

## 1 Introduction

As high-throughput sequencing technologies improve, the analysis of microbial community composition and function has rapidly advanced. Historically, this has mostly focused on taxonomic surveys using a small number of phylogenetically informative genes such as the small subunit of ribosomal RNA (Scholz *et al.*, 2012; Tap *et al.*, 2009). The ability to taxonomically profile communities provided new insights into the role of microbiomes in human health (Cho and Blaser, 2012; Qin *et al.*, 2010), soil ecology (Hultman *et al.*, 2015; Rinke *et al.*, 2013) and environmental remediation (Fierer *et al.*, 2013). Nevertheless, the gene survey approach provides limited functional knowledge because microorganisms with similar or even identical rRNA sequences often differ significantly with respect to genomic content, and therefore may have vastly different functional roles in their environment (Sentausa and Fournier, 2013).

Functional profiling of microbial communities based on Whole Metagenome Shotgun (WMS) sequencing data attempts to catalog the genes present in a community. An inventory of the protein coding functions of a microbial community can be created by either matching the individual reads to annotated reference databases or by assembling the reads and annotating the resulting chromosomal segments (Nagarajan and Pop, 2013). Conventional methods such as BLAST are robust but computationally intensive and techniques for rapidly mapping DNA reads to annotated reference genes fail when the references within the curated databases diverge moderately from DNA sequences of homologous genes in the metagenome sample. To mitigate these challenges, researchers often turn to metagenome assembly and subsequent annotation, which has profound shortcomings, such as chimeric assembly of closely related sequences, strong bias toward abundant organisms, and substantial human and computer resource requirements (Nagarajan and Pop, 2013; Scholz *et al.*, 2012). Therefore, current approaches are not sufficient to satisfy the requirements of researchers attempting to understand functional metagenomics.

**Fig. 1.**
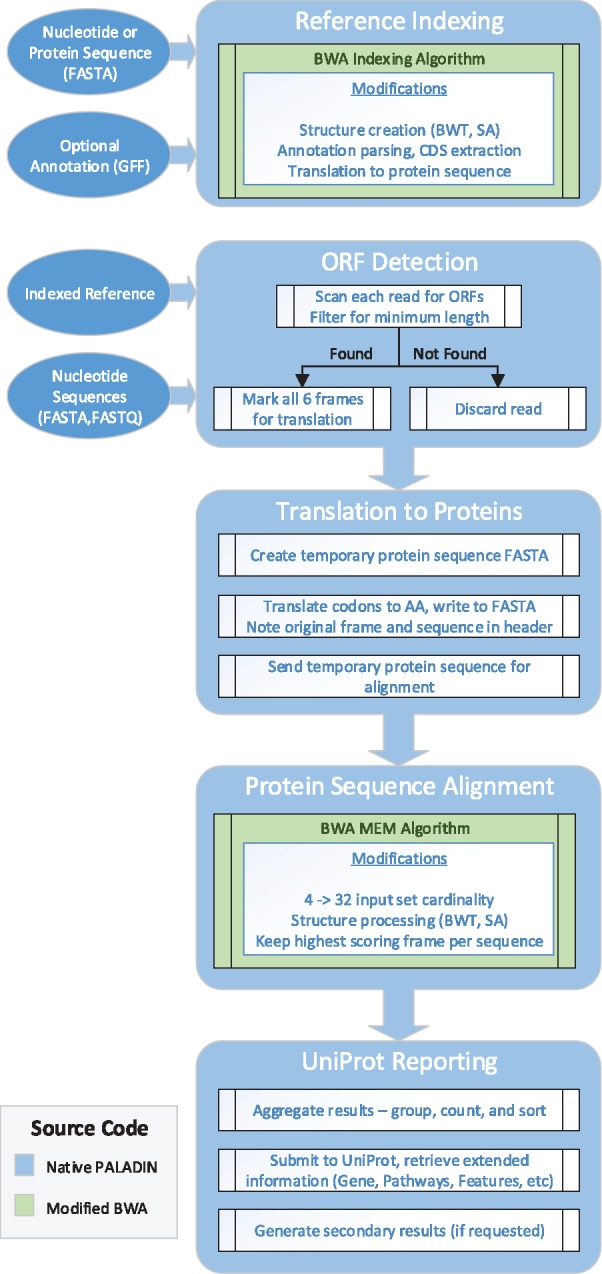
PALADIN internal pipeline, outlining each step in the indexing and alignment process, modifications made to the BWA source, and options for file input. Shading indicates if native PALADIN code or modified BWA code. See Supplementary Figure 5 for output options and further pipeline details

**Fig. 2.**
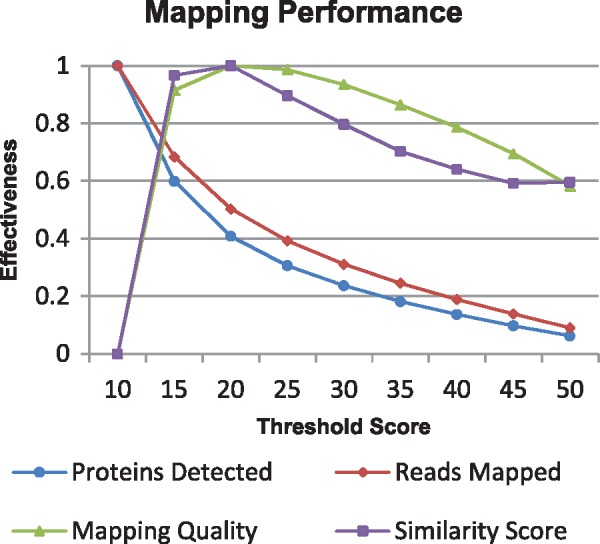
PALADIN performance for the given range of minimum score threshold values. Tests were performed by aligning the generated read set against the UniProt SwissProt reference database. Performance is measured by normalizing the percent of reads mapped, the similarity score, the average mapping quality and the number of unique proteins detected. As the maximal generalized performance of all four metrics centers around the threshold score of 15, this was used as the default parameter value in PALADIN. Variations to the score threshold were shown to have a consistently larger impact on performance than other alignment parameters

To improve the sensitivity and performance of metagenomic functional profiling, we developed PALADIN—software that modifies and extends the popular mapping tool, BWA (Li and Durbin, 2009), to align in protein space rather than nucleotide space. In brief, PALADIN identifies and translates six possible open reading frames within each read, and maps these translated DNA sequences to a protein reference allowing for rapid identification of functional homologies. Specifically, by mapping fully in protein space, this method takes advantage of the general conservation of amino acid sequences compared to the underlying DNA sequences. In this manner, the speed of BWA's local alignment algorithm using super-maximal exact matches is maintained, while the effective results now favor identification of function over taxonomy. Here we demonstrate the practical application of this modified alignment algorithm using large scale WMS datasets. PALADIN reports mappings in standard SAM format, and can generate a tab-delimited file from which additional information can be obtained, including protein abundance, gene ontology and mapping quality (Figs 1 and 2).

## 2 Methods

To evaluate the overall performance of our protein space read mapper, we established three primary goals. The first was to verify base functionality by correctly mapping a positive control simulated read set generated from six bacterial genomes. Secondly, to investigate and contrast the effect of protein sequence alignment against comparable sequences in nucleotide space, two types of comparisons were performed: degenerate nucleotide mapping (see Supplementary Note 1) using NovoAlign (http://www.novocraft.com), a read mapper documented to align against ambiguous IUPAC characters in the reference; and standard nucleotide mapping using BWA, the tool sharing the same codebase as PALADIN, thereby reducing potential variables. For these tests, the well-curated UniProt Swiss-Prot database was used as the reference, with entries corresponding to the six bacterial genomes removed to ensure mapping was performed on function and not exact sequence. Lastly, after accuracy and efficiency of protein mapping was established against both styles of nucleotide mapping, we evaluated the performance of PALADIN compared with the popular conventional protein alignment algorithm, BLASTX, as well as three more recent alternatives.

### 2.1 Establishing a positive control

We first generated typical 250 basepair long paired-end reads using the standard Illumina error model for six well-annotated bacterial genomes (*Pseudomonas fluorescens*, *Escherichia coli*, *Acidovorax avenae*, *Micrococcus luteus*, *Halobacillus halophilus* and *Staphylococcus epidermidis*) using the read simulation package ART (Huang *et al.*, 2012). The reads for the six genomes were pooled to create a mock-metagenomic read dataset (see Supplementary Note 2). As ART includes position indices of the generated read relative to the original reference within each FASTQ header, scripts were employed to link each read to its corresponding CDS entry within the respective reference genome's GFF annotation, then record the corresponding GO terms for each entry using cross-referenced identifiers. This allowed us to definitively determine if a read was correctly mapped, generate the ontology terms for each associated gene, and populate the read graph for Jaccard similarity calculations performed in subsequent tests.

With this source information pre-established, we then mapped the combined reads using PALADIN against a reference consisting of the protein sequences corresponding to the CDS entries of the six original genomes. BWA and NovoAlign were also compared in this fashion, instead using the nucleotide sequences (standard and degenerate, respectively) corresponding to the CDS entries of the six original genomes as the reference.

### 2.2 Evaluating accuracy of functional mapping

By leveraging our prior knowledge about the genes corresponding to the simulated reads, we evaluated the functional accuracy of mapping using four metrics—percentage of reads mapped, average mapping quality, Jaccard similarity coefficient (Jaccard, 1901) and the number of unique proteins found in Swiss-Prot. To calculate the similarity coefficient, reads and their corresponding aligned targets were assigned functionality using the standardized Gene Ontology (GO) language. Each GO term represents a vertex within a graph formation where conditional edges join parent terms tracing back to one of three root vertices: biological processes, molecular function and cellular component. For each read and its matching Swiss-Prot entry, graphs were constructed by the GO term assignments directed back to their respective root vertices. Via this method, distance and overlap between two graphs quantify the functional similarity between two corresponding sets of GO terms. The ratio of the intersection and the union of both graphs was then used to determine the Jaccard similarity coefficient as an alignment accuracy metric (see Supplementary Note 3). Lastly, the number of unique proteins was determined based on each distinct Swiss-Prot ID the reads mapped to, filtered by a minimum mapping quality score.

While mapping mock reads to the well annotated Swiss-Prot database allows us to assay accuracy via the use of GO-term similarity, it is a relatively small dataset with limited representation of both functional and taxonomic breadth. A more ideal reference would be the UniRef90 database which contains taxonomically diverse sequences clustered at 90% sequence identity. In a process identical to above, we mapped DNA reads of three published WMS datasets from different environments to a large subset of proteins within the UniRef90 using PALADIN, and mapped these reads to the nucleotide sequences corresponding to this protein set using BWA. Results were then analyzed for all metrics except similarity, which requires prior knowledge of all genomes within the read set.

### 2.3 Comparing performance

To contrast the differences in computational efficiency between PALADIN and conventional protein alignment tools, we mapped a dataset consisting of nearly 240 000 000 reads, and one consisting of 1 000 000 reads against the UniRef90 database with PALADIN using 28 cores on a high-end workstation. Using the smaller read set, tests were run using three recently developed BLAST alternatives: RAPSearch2 (Zhao *et al.*, 2012), DIAMOND (Buchfunk *et al.*, 2015) and Lambda (Hauswedell *et al.*, 2014). Due to obvious constraints in execution time, we then further extracted 8000 of these sequences and performed an alignment with BLASTX (Altschul *et al.*, 1990). Each test was performed using the same hardware environment, thread count and resource availability. Because local alignment algorithms execute in linear time, effective alignment efficiency was calculated for each solution by first subtracting the time involved in loading the indexed reference, then dividing the remaining time by the number of reads processed.

To ensure performance was evaluated at equivalent levels of sensitivity and accuracy, two metrics were calculated for each aligner. The first metric linked each read PALADIN successfully aligned, filtered by a mapping quality threshold, to the corresponding alignment reported by the comparison tool, noting the percentage of where both detected proteins were in consensus. This ensured that reads mapped with high probable accuracy matched between both tools. The second metric demonstrated the relative lower quality of the remaining alignments made by the comparison tool that were either marked as low quality by PALADIN, or not successfully aligned by PALADIN due to low scores or ORF detection removal. While a direct comparison of mapping quality values was not possible as no tested aligner populates the MAPQ field in the SAM output, the bit score was instead employed to represent quality. The mean bit score was noted for all consensus alignments, and for all alignments PALADIN did not map, with the difference of the two representing the base-2 magnitude of difference in quality. Lastly, this value was converted into a base-10 order-of-magnitude score, illustrating the lower alignment quality of reads left unmapped by PALADIN:

(1) $$S=\log2^{\left(a-b\right)}$$

where *a* and *b* represent the respective mean bit scores of the consensus reads and the unaligned reads. In the case of unaligned reads, only the highest scoring alignment per read was aggregated in calculating the average.

### 2.4 Applicability of prior work

In addition to the applications tested, many alternative alignment tools with documented efficiency gains were considered, but did not meet the criteria for comparison. The most common attribute restricting read mappers was an inability to query nucleotide sequences against degenerate or protein references. Similarly, though many BLAST competitors are available, most do not allow for directly contrasting performance. USEARCH and UBLAST (Edgar, 2010) are only provided for free as 32-bit applications, and are unable to index the UniRef90 due to database size. VSEARCH (Rognes *et al.*, 2016), documented as a competitor to USEARCH, does not support amino acid sequences. BLAT (Kent, 2002) does not support the BLASTX combination of 6-frame DNA queries against protein references. PAUDA (Huson and Xie, 2014) internally maps nucleotide sequences via Bowtie2 (Langmead and Salzberg, 2012) and does not perform true protein alignment (see Supplementary Note 4).

## 3 Results

### 3.1 Nucleotide alignment versus protein alignment

As expected, when mapping reads to the genomes they were derived from during the positive control phase, PALADIN, NovoAlign and BWA all map with high accuracy, yielding respectively 93.39%, 91.65% and 86.23% of these reads correctly mapped (Table 1). Similarly, all three aligners reported high mapping quality scores, further suggesting correct alignment. While both PALADIN and BWA also aligned the majority of reads, the poor performance of NovoAlign for this metric appears to suggest that while it accepts degenerate bases in the reference, a penalty is incurred during alignment due to ambiguous IUPAC characters.

**Table 1.** Establishing a positive control

	BWA	NovoAlign	PALADIN
Reads mapped %	96.02	36.39	98.00
Correctly mapped %	86.23	91.65	93.39
Mapping quality (60)	58.67	59.84	59.11
Detected proteins	20461	21889	22127

Positive control was established by aligning the simulated reads against the coding regions of the original six test genomes. Percentage of reads correctly mapped and mapping quality were used to demonstrate algorithm and method correctness. Quality scores are calculated using the Phred scale, with 60 representing the highest level of confidence.

When we tested the ability of PALADIN to map mock-metagenomic reads to the Swiss-Prot database, it detected 7855 unique proteins compared to 6314 (BWA) and 2265 (NovoAlign). Additionally, both percent of reads mapped and similarity scores were higher in PALADIN (Table 2). While NovoAlign did score higher in average mapping quality, this was aggregated across a dramatically smaller percentage of mapped reads, at 0.56%. Similar patterns in detected protein counts and mapped percentages were also found in all three empirical sets (Table 3). These results suggest that mapping in protein space as implemented in PALADIN is a more accurate method of establishing a functional profile than existing nucleotide solutions.

**Table 2.** Mapping efficiency against filtered Swiss-Prot

	BWA	NovoAlign	PALADIN
Reads mapped %	19.79	0.56	25.65
Similarity index	0.81	0.81	0.85
Mapping quality (60)	25.21	54.51	25.88
Detected proteins	6314	2265	7855

Mapping efficiency against the filtered Swiss-Prot database using simulated reads. Detected proteins were filtered for reads with 20 or greater mapping quality scores. Results showed an improvement in both quantity and functional accuracy when aligning in protein space with PALADIN.

**Table 3.** Detected proteins mapped against the UniRef90

Type	Project	BWA	PALADIN
Lung	Cystic Fibrosis Metagenome	60 296	40 251*
Gut	HMP Core Microbiome	175 448	190 947
Soil	Merlot Microbiome	7921	11 792

Number of proteins detected for three empirical read sets mapped against the UniRef90: Lung (BioProject:PRJNA71831), Gut (BioSample:SAMN00037421) and Soil (MG-RAST:4520320.3), when filtered for reads with 20 or greater mapping quality scores. Note, the lower performance of PALADIN for the lung set is a result of the proteins being underrepresented in the filtered version of the UniProt90. Mapping against the full UniRef90 yielded 66 592 proteins detected.

### 3.2 PALADIN versus protein aligners

Lastly, the difference in performance between PALADIN and the four conventional protein alignment tools was especially significant. In regard to the smaller dataset, PALADIN completed execution in the least amount of time, mapping 1 000 000 reads in approximately 7 min (Table 4). The next fastest, DIAMOND, finished execution in nearly 52 min. During the full test, PALADIN completed mapping the set of 240 000 000 reads over a period of 31 h for an efficiency of approximately 128 000 reads/min. Conversely, BLASTX aligned its subset of 8000 reads in 8.5 h with an efficiency of about 16 reads/min. Given the linear time complexity associated with BLASTX, we estimate that an execution run against the full dataset would take about 29 years, approximately 8000 times longer than PALADIN.

**Table 4.** Performance comparison for large read sets

Read Count	Aligner	Effective Time (HMS)	Aligns/min
1 000 000	PALADIN	00:07:10	139 535
1 000 000	RAPSearch2	32:54:59	506
1 000 000	DIAMOND	00:51:42	19 342
1 000 000	Lambda	04:46:23	3492
240 000 000	PALADIN	31:15:02	127 998
240 000 000	BLASTX	250 000:00:00	16

Performance evaluation of the three applicable BLAST competitors was performed against a 1 000 000 read subset of the full 240 000 000 read set. To estimate the linear portion of alignments per second, the effective time reflects the difference between total time and the constant setup time of loading the indexed reference. Due to obvious time constraints in the case of the BLASTX comparison, alignment rate was calculated using an 8000 read subset, from which the effective time was estimated. For this final test, the PALADIN was given the entire 240 000 000 set as a query. In both tests, PALADIN outperformed all other alignment tools.

Accuracy tests also yielded consistent results between PALADIN and the conventional protein aligners (Table 5). For each read PALADIN mapped with high quality, all four tools aligned to the matching protein consistently, with consensus ranging from 96% to over 99% (see Supplementary Note 5). For reads reported by PALADIN as mapping with low quality, or those left unmapped, the four tools produced alignments with orders-of-magnitude worse mean bit scores than the consensus alignments, ranging up to a magnitude of 14.33 in the case of Lambda. Taken together, these metrics illustrate PALADIN's significant performance gains while continuing to maintain comparable accuracy with other protein alignment tools.

**Table 5.** Accuracy comparison for large read sets

Aligner	Consensus %	Bit Difference	Quality Magnitude
RAPSearch2	99.34	n/a*	n/a*
DIAMOND	96.36	45.38	13.66
Lambda	98.85	47.62	14.33
BLASTX	99.23	20.60	6.20

Accuracy evaluation of the four protein alignment tools, calculated against the read sets outlined in Table 4. Consensus percentage indicates the portion of PALADIN mapped reads (filtered for a mapping quality score of 50) that matched the subject protein of the compared tool's alignment. Quality magnitude, calculated via the mean bit score difference, notes the significant decrease in alignment quality of reads left unmapped by PALADIN. Note, this calculation could not be performed on the RAPSearch2 results, as a software bug prevented writing read headers to the alignment report.

### 3.3 Limitations

Though results suggest that PALADIN is more effective than nucleotide read mappers at constructing functional profiles, this is inherently at the cost of taxonomic identification sensitivity. The effect of translating nucleotides to corresponding amino acids results in a loss of information between synonymous sequences, increasing ambiguity in taxonomic differentiation. Secondly, as this underlying strategy employs pairwise alignment and relies on amino acid sequence similarity, PALADIN is not well-suited for detecting remote homologies where structural similarity exists between highly divergent sequences.

## 4 Implementation

In order to leverage the stability and extensive capabilities of a proven application, we elected to make use of the BWA codebase for PALADIN's underlying alignment framework(Fig. 1). In particular, the Super-Maximal Exact Matches (SMEM) algorithm was extended into protein space via modification of many of the internal routines, including the Burrows-Wheeler Transform construction process, substitution matrix generation, mapping quality probability calculations, data packing, character occurrence counting, parameter values and other related data structures. The resulting code allows both seeding and Smith-Waterman extension to operate on an input set consisting of amino acid IUPAC characters, and not the standard nucleotide character set.

Additionally, the holistic end-user experience of PALADIN was examined, and a number of novel features were introduced to accommodate a design that allows for all stages of the internal protein alignment pipeline to occur seamlessly without manual intervention. First, we added ORF detection prior to the alignment phase. During this stage, PALADIN searches all six frames of each read sequence for the existence of stop codons within a minimum length threshold, as specified by an absolute length or relative percentage of total length. If no stop codon is found within at least one frame, the read is considered to originate from a coding region. Reads with stop codons present in all frames are discarded without further analysis for efficiency. Surviving reads are then translated for each of the six possible reading frames into the corresponding amino acid sequences, and sent for alignment. The highest scoring frame's protein sequence is kept for final output, and the five remaining are discarded. By post-processing all frames in this manner, the cascading effect of potential frameshift mutations and sequencing errors is mitigated in cases where indels appear early or late in the read, allowing the majority of the frameshifted sequence to match the reference. In scenarios where repeated indel history has led to multiple framing errors, lower alignment scores are appropriate as these sequences are less likely functionally similar to the reference sequence in question.

Following the alignment phase PALADIN produces a standard Sequence Alignment Map (SAM) output along with a detailed tab-delimited report derived from the reference database (SwissProt and UniRef90 are currently supported). The software aggregates and calculates abundance, via sorting and grouping successfully aligned reads against the mapped UniProtKB ID, calculates average mapping quality for each entry, then batch submits these entries to the UniProt REST API (see Supplementary Note 6), requesting additional information pertaining to each identified Uniprot entry, including protein, gene, pathway features, gene ontology, organism, reviewed status, database cross-references, etc. The returned results are compiled into a tab-delimited report, and are provided alongside the accompanying SAM file.

Should the user wish to make use of a reference other than a UniProt database, PALADIN also supports indexing three types of references. A nucleotide reference and accompanying GFF annotation may be provided, in which case the software extracts the CDS features to create the index. A transcriptome nucleotide reference may be provided, in which all sequences are treated as coding when creating the index. Lastly, a protein reference may be provided, in which all sequences are again treated as coding when creating the index.

Because PALADIN is constructed from the BWA codebase, it supports many of the same alignment parameters and corresponding command line arguments, including seed length, scoring related parameters and SAM output options. However, since alignment in protein space operates under a different statistical environment than nucleotide space, some of the default values, such as seed size, minimum threshold score (Fig. 2), clipping penalty and gap opening penalties were changed in response to the higher local performance maxima detected during extensive parameter testing (see Supplementary Note 7).

## 5 Conclusion

In summary, we present PALADIN, a tool for accurate functional characterization of metagenomic samples that improves over existing nucleotide and protein alignment solutions, demonstrating an orders-of-magnitude increase in speed when compared to the latter. This significant improvement in efficiency affords researchers unprecedented opportunity to gain detailed and novel insight into microbial communities. Additionally, by constructing this approach upon a widely used alignment algorithm, reliability and usability are inherently increased, which promotes faster adoption and easier incorporation into existing pipelines. Finally, the reduction in required computational resources creates a more cost-effective solution, thereby increasing viability of analysis capabilities in environments where economic pressures are present. Given these aspects, PALADIN may potentially aid in any number of evolving fields that depend on functional characterization, including personalized medicine, biodefense, environmental remediation, transcriptomics and the study of emerging pathogens.

## Supplementary Material

Supplementary Data
