# Supplementary material for: PALADIN: protein alignment for functional profiling whole metagenome shotgun data
Source: Bioinformatics. 2017 Jan 31;33(10):1473–8. doi: 10.1093/bioinformatics/btx021 (PMC5423455; doi:10.1093/bioinformatics/btx021)
Supplement: Supplementary Data [file btx021_supp.zip › btx021-suppl_data/PALADIN - Supplementary.zip!/Supplementary - Primary - Bioinformatics.pdf]

PALADIN Supplementary

Note 1. Creating degenerate references

In order to compare the effectiveness of synonymous codon alignment as represented by amino acid characters (protein alignment) with ambiguous nucleotide characters (degenerate nucleotide alignment), NovoAlign was chosen for its support of all IUPAC characters. To create the degenerate versions of the nucleotide references for use with NovoAlign, an in-house script was executed against both the six bacterial genomes, as well as the nucleotide mined version of the Swiss-Prot database. In the case of the bacterial genomes, the GFF annotation files were parsed for CDS entries, whereas the entire sequence of each Swiss-Prot entry was treated as coding. Coordinates, direction, and phase were extracted from each CDS entry to calculate each coding region within the genome. For each region, the third codon position was replaced with the corresponding ambiguous IUPAC character corresponding to pre-translated possibilities resulting in the effective amino acid. In the case of overlapping coding regions, the most ambiguous IUPAC character still meeting the requirements of the resultant amino acid was chosen. To accomplish this, replacement occurred across two phases. Phase 1 masked all effected positions within the genome, while phase 2 actually performed the replacement, calculating for multiple, overlapped masks.

Note 2. Simulating metagenomic dataset

We generated paired-end reads *in silico* for six well-annotated bacterial genomes using ART– *Pseudomonas fluorescens* F113 (NC_016830.1), *Escherichia coli* str. K-12 substr. MG1655 (CP009685.1), *Acidovorax avenae* subsp. avenae (CP002521.1), *Micrococcus luteus* NCTC 2665 (CP001628.1), *Halobacillus halophilus* DSM 2266 (HE717023.1), and *Staphylococcus epidermidis* ATCC 12228 (CP000029.1). We chose read length of 250 bp, fragment size of 1000 nucleotides with a 10% standard deviation, and 100X coverage. The default Illumina sequencing system profile ("HS25 - HiSeq 2500") was used for the quality and error model. The forward reads for the six genomes were pooled into a metagenomic dataset.

As ART includes both start and end position indices of each generated read, these were used to lookup the corresponding CDS entry within the respective reference genome's GFF annotation for accuracy testing. Reads spanning multiple CDS entries were ignored during this stage of testing.

Note 3. Jaccard Similarity Coefficient

The Jaccard index was calculated for ontological similarity using the standard method:

$$J(A,B) = \frac{|A \cap B|}{|A \cup B|} = \frac{|A \cap B|}{|A| + |B| - |A \cap B|}$$

Where sets A and B represent the sets of vertices in the gene ontology graphs generated for the mapped proteins and the organism's known proteome, respectively. In this manner, a normalized score was calculated representing the similarity between the two graphs.

Note 4. Evaluating PAUDA

In addition to RAPSearch2, DIAMOND, Lambda, and BLASTX, we also considered evaluating PAUDA (Protein Alignment Using a DNA Aligner). As PAUDA internally employs a third-party nucleotide read mapper, and does not directly align amino acid characters, we initially had concerns regarding comparability to the conventional protein aligners. To investigate if inclusion was appropriate, we performed the tests as outlined in section 2.3, evaluating both accuracy and performance. Executing PAUDA using the million read set completed in 00:52:03, comparable to the next fastest aligner to PALADIN, DIAMOND. However, the accuracy tests demonstrated a significantly reduced consensus percentage with PALADIN, with only 59.12% of PALADIN's primary alignments matching any PAUDA hits for the corresponding read. The previously lowest scoring protein aligner, DIAMOND, had a consensus of 96.36%, 37.24% more than PAUDA.

Due to the magnitude of the difference in consensus, we wanted to account for the possibility that PAUDA's results were higher quality than PALADIN's. In such a scenario, PAUDA's results would match the other protein aligners more often than PALADIN. To test this possibility, we developed a script that compared the primary alignments of each protein aligner against those of PALADIN and PAUDA. For queries with hits across all three tools, the script noted when the alignment agreed with PALADIN's alignment, and when it agreed with PAUDA's alignment. DIAMOND, Lambda, and BLASTX each agreed with PALADIN to a much higher degree than PAUDA, with respective normalized averages of 89.44% and 75.65%. Given this performance in contrast to the other protein aligners, and the additional investigative testing outside of the scope of the paper, we have instead confined the results to the supplementary materials only.

Note 5. Verifying the positive control against protein aligners

As the difference in consensus between PALADIN and the protein alignment tools ranged up to 4% for reads PALADIN mapped with a quality of at least 50, we felt it prudent to further analyze this discrepancy and confirm if it was the result of the protein alignment tools performing poorly for the non-consensus reads. To eliminate the uncertainty associated with empirically obtained read sets, we instead performed accuracy testing using the positive control set, similar to the tests performed with the nucleotide read mappers. Since the protein aligners mapped all reads present in the set, correctly mapped reads were the only informative metric. Additionally, to ensure a comparison with identical evaluation methodology to the read mappers, only the primary hit for each query was considered. Results indicated PALADIN maps the control set more accurately than the protein aligners, confirming the discrepancy was indeed due to poor performance for the non-consensus reads.

Table 1. Comparing positive control for protein aligners

Aligner	***Correctly mapped %***
PALADIN	93.39
RAPSearch2	n/a*
DIAMOND	90.52
Lambda	90.34
BLASTX	87.67

The positive control evaluations outlined in primary Table 1 were repeated for the protein aligners to investigate the consensus discrepancy noted in primary Table 5. Note, this calculation could not be performed on the RAPSearch2 results, as a software bug prevented writing read headers to the alignment report.

Note 6. Uniprot REST API

The UniProt report is a comma delimited file generated by performing batch downloads of associated information from the UniProt website via their RESTful web services (http://www.uniprot.org/help/programmatic_access). UniProt KBIDs are transmitted in batches of 10,000 per the suggestions of the UniProt Consortium to reduce the probability of server timeouts and workload. In the event of a server error or unexpected event, PALADIN will retry the submission five times before aborting.

Note 7. Parameter evaluation

Sweeps of parameter values were tested across seed length, minimum score, mismatch penalty, affine gap penalties, and clipping penalty. Tests were generally performed across a large range, then refined across smaller ranges once patterns had been established. Performance was considered for mapped read percentage, mapping quality, similarity, and number of proteins detected. Those parameter value with high scoring quality and proteins detected were selected.

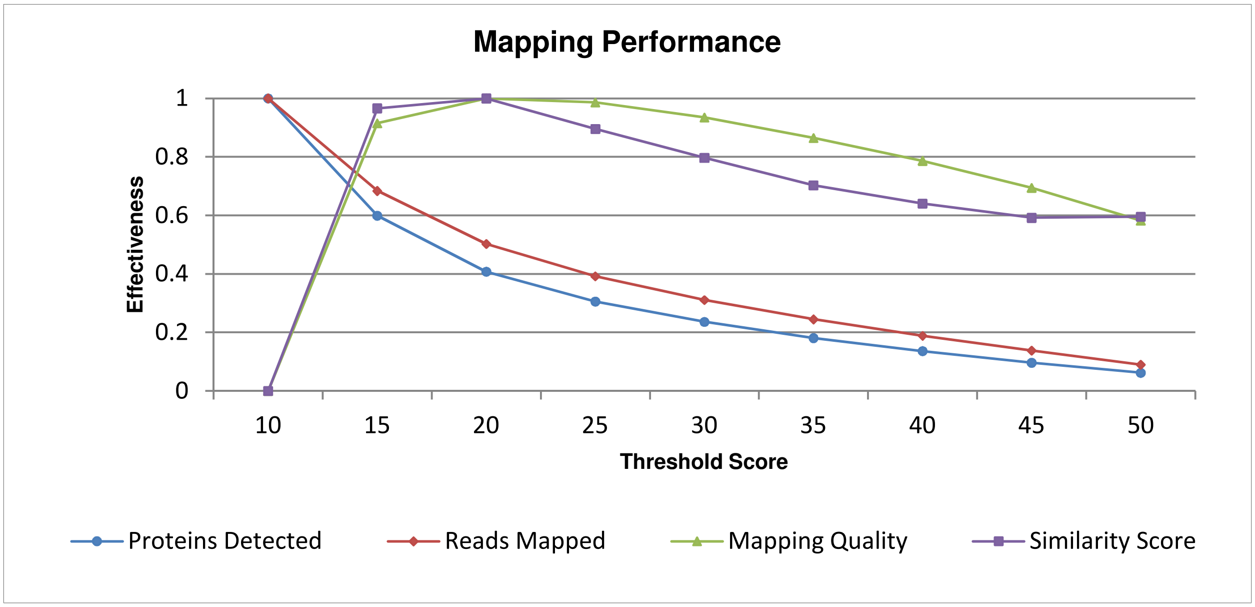

Fig. 1. Comparison of PALADIN alignment performance results for the given range of minimum threshold score values. Tests were performed by aligning the in silico generated read set against the UniProt SwissProt reference database, specifying the "-T" option with each respective threshold value per iteration. Alignments with scores lower than this value are discarded by PALADIN during execution. Performance is measured by the percent of reads mapped, proteins detected, mapping quality, and similarity. While the first two metrics present a generally inverse relationship to the last two, the maximal performance centers around a threshold score of 15.

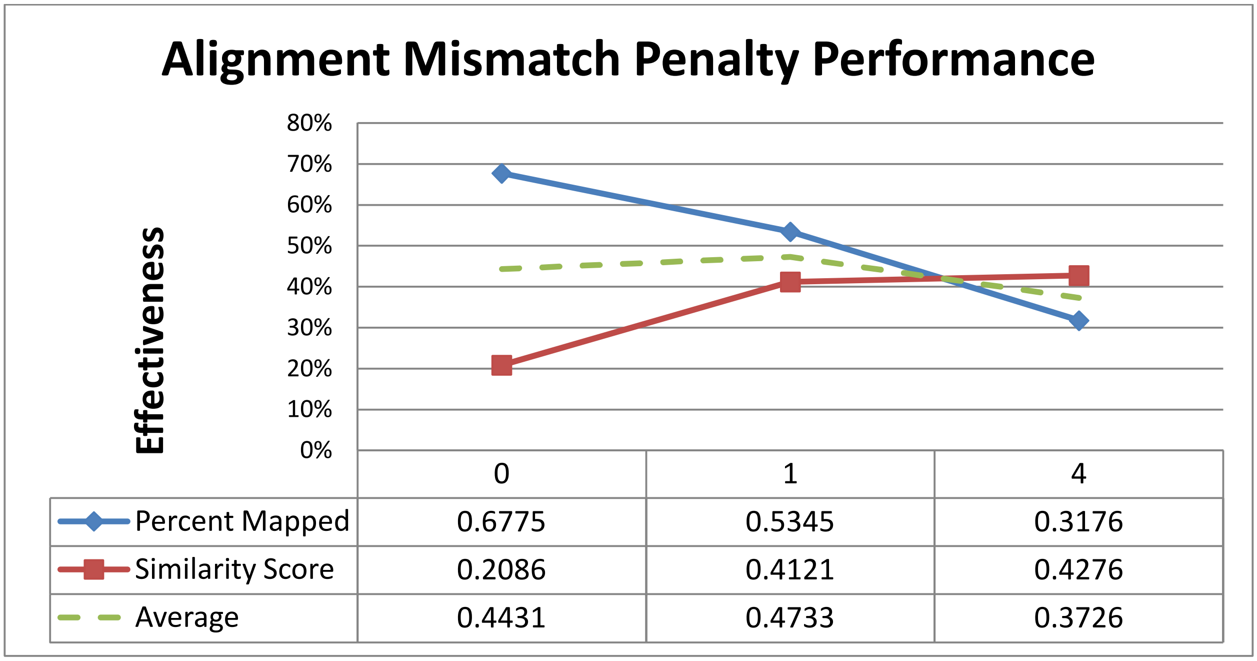

	0	1	4
Percent Mapped	0.6775	0.5345	0.3176
Similarity Score	0.2086	0.4121	0.4276
Average	0.4431	0.4733	0.3726

Fig. 2. Comparison of PALADIN alignment performance results for the given range of mismatch penalty values. Tests were performed by aligning the in silico generated read set against the UniProt SwissProt reference database, specifying the "-B" option with each respective mismatch penalty value per

iteration. Amino acids in the read sequence not matching the reference incur this negative penalty from the total score by PALADIN during execution. Performance is measured by the percent of reads mapped, the similarity score, and the average of these two values.

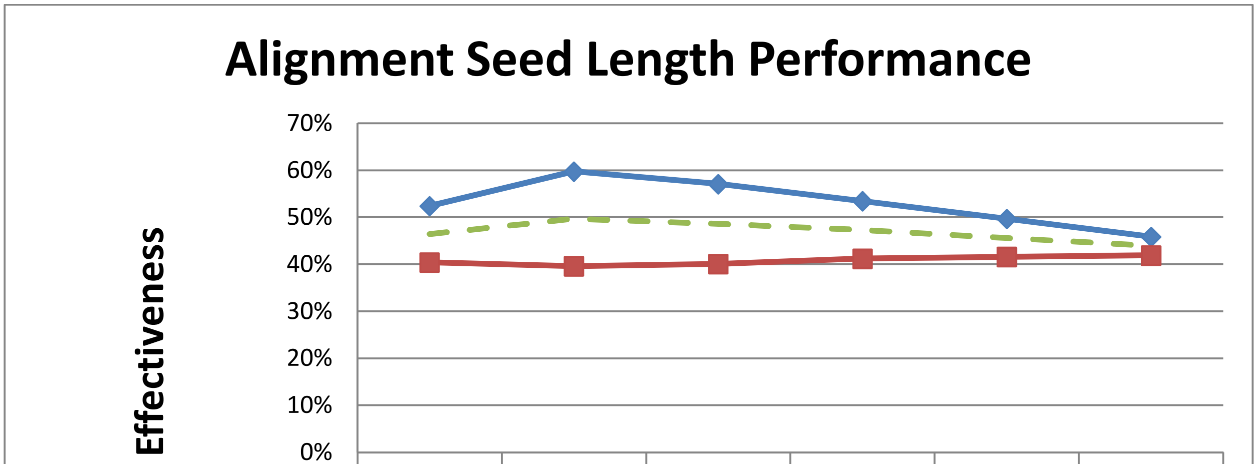

	8	9	10	11	12	13
Percent Mapped	0.5241	0.5975	0.5708	0.5345	0.4967	0.4586
Similarity Score	0.4041	0.3962	0.4008	0.4121	0.4159	0.4189
Average	0.4641	0.4968	0.4858	0.4733	0.4563	0.4388

Fig. 3. Comparison of PALADIN alignment performance results for the given range of seed lengths. Tests were performed by aligning the in silico generated read set against the UniProt SwissProt reference database, specifying the "-k" option with each respective seed length value per iteration. Amino acid substrings shared by the read sequence and reference shorter than this value are discarded by PALADIN during execution. Performance is measured by the percent of reads mapped, the similarity score, and the average of these two values.

Note 8. Script usage

The "mappedCDS.py" Python script was used to create a tab delimited file that linked ART generated reads that were fully contained within a CDS entry to that CDS entry. This script was used in conjunction with both the "similarity_tester.py" and "matchCompareOrig.py" scripts. The GFF files associated with the 6 test genomes must be present in the same directory when executing.

mappedCDS.py <sam file> <starting index (5 for PALADIN, 2 for others)>

The "degenerateFasta.py" Python script was used to create degenerate versions of references, used in testing NovoAlign. This script supports two modes of operation - the first takes a FASTA and a corresponding GTF/GFF annotation, and extracts the sequences based on the CDS entries. This was the method used for the six original genomes. The second mode (using the "--all" switch) considers the entire sequence coding and ignores the GTF/GFF file - this was used for the nucleotide version of the SwissProt database.

degenerateFasta.py --fasta <fasta file> --gtf <gtf file> --output <degenerate reference output> [--all]

The "similarity_tester.py" Python script was used to generate similarity scores from a given SAM file when aligned using sequences generated from ART from the six original genomes. The first argument of the script specifies if the SAM file was generated by PALADIN, or bwa/novoalign. It sends output scores to stdout.

similarity_tester.py <paladin|bwa> <sam file>

The "paudaParse.py" Python script was used to parse and convert the BLASTX-like output of PAUDA over to a tab delimited file for processing in the "matchCompareBlast.py" and "matchCompareMultiple.py" scripts.

paudaParse.py <pauda output> <mode>

The "matchCompareOrig.py" Python script was used to calculate reads that mapped correctly during the positive control tests.

matchCompareOrig.py <sam file>

The "matchCompareBlast.py" Python script was used to calculate the consensus percentage and relative bit score difference magnitude between PALADIN and the comparison protein aligner. DIAMOND and Lambda outputs were specified as SAM files (3rd parameter "sam"), BLASTX made use of the full report (3rd parameter "blast"), and PAUDA output was first converted using "paudaParse.py" (3rd parameter "pauda"). Since RAPSearch2 appears to contain a bug that omits some read headers from the ALN file, a special method was employed that skips calculating the second metric (third parameter "rap").

matchCompareBlast.py <paladin SAM file> <aligner output file> <sam|blast|rap|pauda>

The "matchCompareMultiple.py" Python script was used to compare the alignments of each protein aligner against both PALADIN and PAUDA, calculating the percentage of agreement for each. DIAMOND and Lambda outputs were specified as SAM files (4th parameter "sam"), and BLASTX made use of the full report (4th parameter "blast"). The PAUDA output was first converted using "paudaParse.py".

matchCompareMultiple.py <paladin SAM file> <PAUDA output> <aligner output file> <sam | blast>

Note 9. Program usage

The following is a quick guide for using PALADIN. For most up-to-date usage and program source, please visit: https://github.com/twestbrookunh/paladin

PALADIN

Protein **AL**ignment **A**nd **D**etection **IN**terface

PALADIN is a protein sequence alignment tool designed for the accurate functional characterization of metagenomes.

PALADIN is based on BWA, and aligns sequences via read-mapping using BWT. PALADIN, however, offers the novel approach of aligning in the protein space. During the index phase, it processes the reference genome's nucleotide sequences and GTF/GFF annotation containing CDS entries, first converting these transcripts into the corresponding protein sequences, then creating the BWT and suffix array from these

proteins. The process of translation is skipped when providing a protein reference file (e.g., UniProt) for mapping. During the alignment phase, it attempts to find ORFs in the read sequences, then converts these to protein sequences, and aligns to the reference protein sequences.

PALADIN currently only supports single-end reads (or reads merged with FLASH, PEAR, abyss-mergepairs), and BWA-MEM based alignment. It makes use of many BWA parameters and is therefore compatible with many of its command line arguments.

PALADIN may output a standard SAM file, or a text file containing a UniProt-generated functional profile. This text file may be used for all downstream characterizations.

INSTALLATION

Dependencies

From a fresh install of Ubuntu, you will need to install `build-essential libcurl4-openssl-dev git make gcc zlib1g-dev`. This should be available on Ubuntu 14.04 using `sudo apt-get install build-essential libcurl4-openssl-dev git make gcc zlib1g-dev`

PALADIN compiles by default on OSX 10.10.x

```
git clone https://github.com/twestbrookunh/paladin.git

cd paladin/

make

PATH=$PATH:$(pwd)
```

SAMPLE COMMANDS

Download and prepare UniProt Swiss-Prot index files.

```
paladin prepare -r1
```

Download and prepare UniProt UniRef90 index files.

```
paladin prepare -r2
```

Index UniProt (or another protein) fasta, if not using the automated `prepare` command

```
paladin index -r3 uniprot_sprot.fasta.gz
```

Align a set of reads using 4 theads. Send the full UniProt report to paladin_uniprot.tsv.

```
paladin align -t 4 -o paladin index input.fastq.gz
```

Align a set of reads using 4 theads. Produce a bam file.

```
paladin align -t 4 index input.fastq.gz | samtools view -Sb - > test.bam
```

Align a set of reads, preferring higher quality mappings over number of proteins detected.

```
paladin align -T 20 -o paladin index input.fastq.gz
```

Align a set of reads, report secondary alignments, and generate UniProt report for both primary and secondary alignments.

```
paladin align -a -o paladin index input.fastq.gz
```

If you're interested in trying this out on a smallish test file, try downloading this one which is from a human lung metagenome study: http://www.ebi.ac.uk/ena/data/view/PRJNA71831

```
#install PALADIN as per above

curl -O
ftp://ftp.sra.ebi.ac.uk/vol1/fastq/SRR117/002/SRR1177122/SRR1177122.fastq.gz

paladin prepare -r1 #unless already done

paladin align -t 4 -o lungstudy uniprot_sprot.fasta.gz SRR1177122.fastq.gz


#look at report file, SAM, etc.
```

OUTPUT

A SAM/BAM file that can be used for any downstream analyses.

A tab delimited UniProt report file.

```
#FORMAT

Count   Abundance Mapping Quality UniProtKB ID  Organism    Protein Names
Genes   Pathway Features    Gene Ontology   Reviewd Existence   Comments
Cross Reference (KEGG)  Cross Reference (GeneID)  Cross Reference (PATRIC)
Cross Reference(EnsemblBacteria)
```

Count: The number of reads mapping to that UniProt entry

Abundance: The percentage of reads mapping to that UniProt entry

Mapping Quality: The average mapping quality for reads mapped to that UniProt entry (Phred scale, max 60)

UniProtKB: The ID containing the Gene short-code and species of origin

ID: The Uniprot code

Organims: The Organims from which the Uniprot ID is derived. Note that one should use this to generate a taxonomic profile of your sample

Protein Names

Genes

Pathway Features

Gene Ontology

Reviewed

Existence

Comments

Cross Reference (KEGG): Corresponding entry in KEGG database (http://www.genome.jp/kegg/)

Cross Reference (GeneID): Corresponding entry in NCBI gene database (http://www.ncbi.nlm.nih.gov/gene)

Cross Reference (PATRIC): Corresponding entry in PATRIC database (http://www.patricbrc.org)

Cross Reference (EnsemblBacteria): Corresponding entry in Ensembl Bacteria database (http://bacteria.ensembl.org)

PALADIN accepts the following parameters (those highlighted in blue are new from BWA):

Preparation

Usage: paladin prepare [options]

Options:

-r <#> Reference Database:

1: UniProtKB Reviewed (Swiss-Prot)

2: UniProtKB Clustered 90% (UniRef90)

-f <ref.fasta> Skip download, use local copy of reference database (may be indexed)

Examples:

paladin prepare -r2

paladin prepare -r1 -f uniprot_sprot.fasta.gz

Indexing

Usage: paladin index [options] <reference.fasta> [annotation.gff]

Options:

-f Enable indexing all frames in nucleotide references

-r<#> Reference type:

1: Reference contains nucleotide sequences (requires corresponding .gff annotation)

2: Reference contains nucleotide sequences (coding only, eg curated transcriptome)

3: Reference contains protein sequences (UniProt or other source)

4: Development tests

Examples:

paladin index -r1 reference.fasta reference.gff

paladin index -r3 uniprot_sprot.fasta.gz

Alignment

Usage: paladin align [options] <idxbase> <in.fq>

Gene detection options:

-b disable brute force ORF detection

-J do not adjust minimum ORF length (constant value) for shorter read lengths

-f INT minimum ORF length accepted (as constant value) [250]

-F FLOAT minimum ORF length accepted (as percentage of read length) [0.00]

Alignment options:

-t INT number of threads [1]

-k INT minimum seed length [11]

-d INT off-diagonal X-dropoff [100]

-r FLOAT look for internal seeds inside a seed longer than {-k} * FLOAT [1.5]

-y INT seed occurrence for the 3rd round seeding [20]

-c INT skip seeds with more than INT occurrences [500]

-D FLOAT drop chains shorter than FLOAT fraction of the longest overlapping chain [0.50]

-W INT discard a chain if seeded bases shorter than INT [0]

-m INT perform at most INT rounds of mate rescues for each read [50]

-e discard full-length exact matches

Scoring options:

-A INT score for a sequence match, which scales options -TdBOELU unless overridden [1]

-B INT penalty for a mismatch [3]

-O INT[,INT] gap open penalties for deletions and insertions [0,0]

-E INT[,INT] gap extension penalty; a gap of size k cost '{-O} + {-E}*k' [1,1]

-L INT[,INT] penalty for 5'- and 3'-end clipping [0,0]

-U INT penalty for an unpaired read pair [17]

-x STR read type. Setting -x changes multiple parameters unless overriden [null]

pacbio: -k17 -W40 -r10 -A1 -B1 -O1 -E1 -L0 (PacBio reads to ref)

ont2d: -k14 -W20 -r10 -A1 -B1 -O1 -E1 -L0 (Oxford Nanopore 2D-reads to ref)

intractg: -B9 -O16 -L5 (intra-species contigs to ref)

Input/output options:

-o STR activate PALADIN reporting using STR as an output file prefix. Files generated as follows:

STR.sam - alignment data (will not be sent to stdout)

STR_uniprot.tsv - Tab delimited UniProt report (normal alignment mode)

STR_uniprot_primary.tsv - Tab delimited UniProt report, primary alignments (all alignments mode)

STR_uniprot_secondary.tsv - Tab delimited UniProt report, secondary alignments (all alignments mode)

-u INT report type generated when using reporting and a UniProt reference [1]

0: Simple ID summary report

1: Detailed report (Contacts uniprot.org)

-g generate detected ORF nucleotide sequence FASTA

-n keep protein sequence after alignment

-R STR read group header line such as '@RG\tID:foo\tSM:bar' [null]

-H STR/FILE insert STR to header if it starts with @; or insert lines in FILE [null]

-j treat ALT contigs as part of the primary assembly (i.e. ignore <idxbase>.alt file)

-v INT verbose level: 1=error, 2=warning, 3=message, 4+=debugging [3]

-T INT minimum score to output [15]

-h INT[,INT] if there are <INT hits with score >80% of the max score, output all in XA [5,200]

-a output all alignments for SE or unpaired PE

-C append FASTA/FASTQ comment to SAM output

-V output the reference FASTA header in the XR tag

-Y use soft clipping for supplementary alignments

-M mark shorter split hits as secondary

-I FLOAT[,FLOAT[,INT[,INT]]]

specify the mean, standard deviation (10% of the mean if absent), max

(4 sigma from the mean if absent) and min of the insert size distribution.

FR orientation only. [inferred]

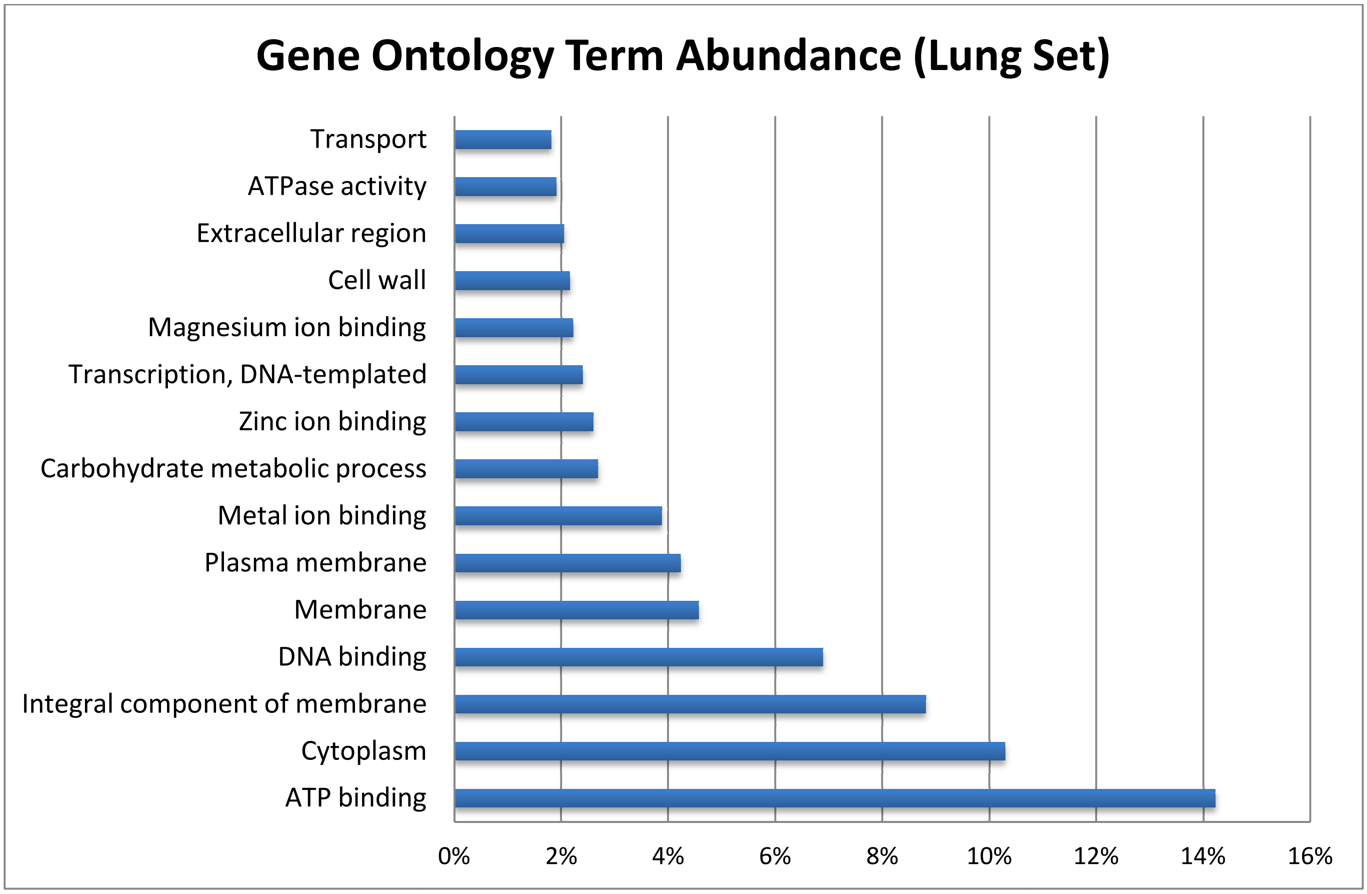

Fig. 4. Top 15 most abundant gene ontology terms present in aligned reads. Test was performed by aligning the CF lung read set against the UniProt Uniref90 reference database. Alignment entries with

multiple GO terms were then split and parsed, and all entries were then weighted by abundance and summated. All data required for graph creation was obtained from the standard PALADIN UniProt output report generated during alignment.

PALADIN Internal Pipeline

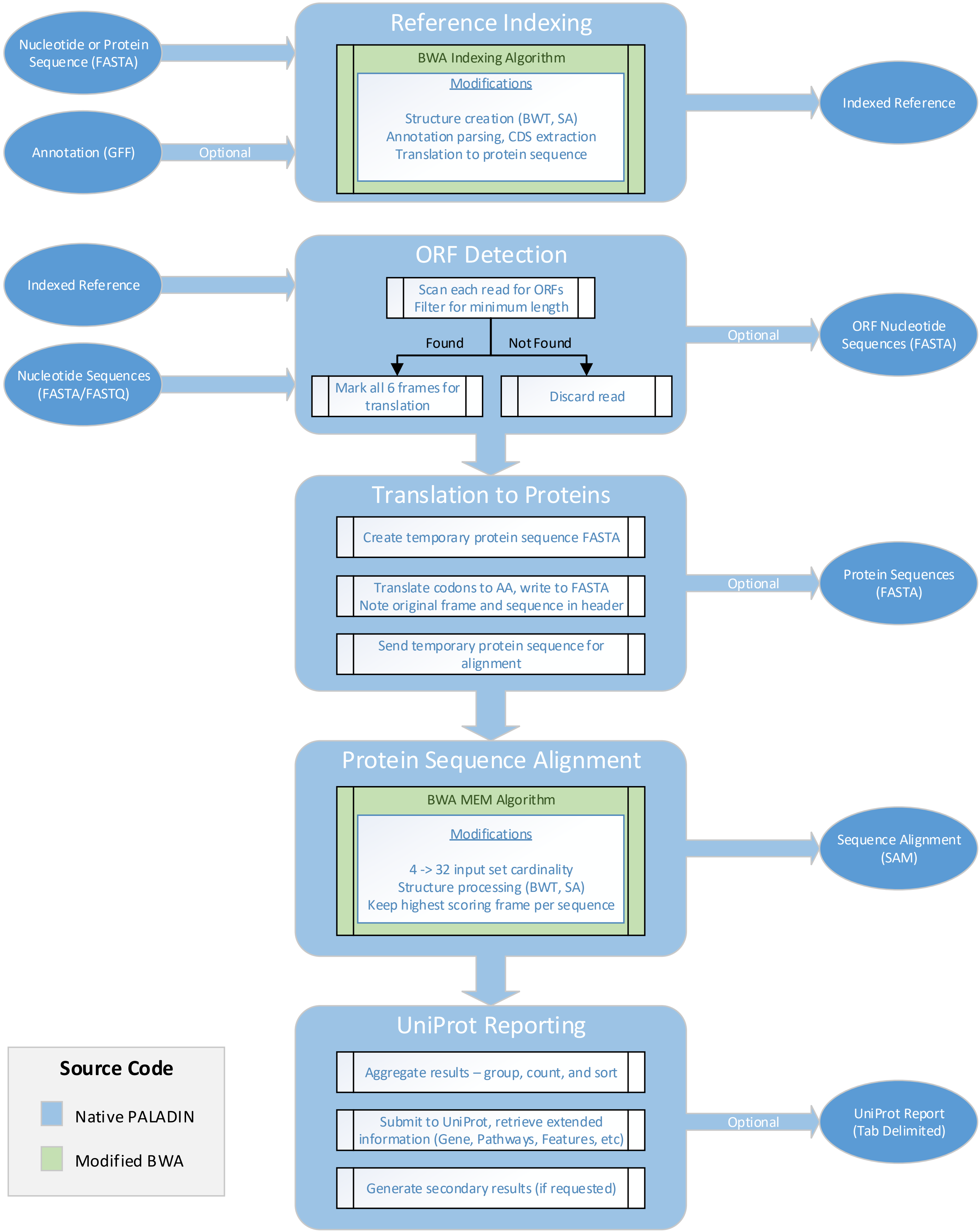

Fig. 5. PALADIN's internal pipeline, detailing each step in the indexing and alignment process, modifications made to the BWA source, and options for file input and output. Steps shaded blue indicate original PALADIN code, while those shaded green indicate original or modified BWA code.

PALADIN's indexing phase consists primarily of the BWA indexing algorithm, with modifications made to the underlying Burrows-Wheeler Transform, suffix array, occurrence counting, and other related structures to operate on an input set of amino acid IUPAC characters. Additionally, functionality related to translation was added, such as parsing CDS features in GFF annotation files, and codon conversions. The output represents an indexed reference in the same fashion as BWA.

The alignment phase has a number of novel features added, including ORF detection, protein translation, and UniProt report generation. ORF detection consists of scanning for the presence of stop codons within a minimum length threshold, then translating and processing those reads where no stop codons are found. Protein translation is performed in the typical manner across all 6 reading frames with all resulting sequences sent for alignment. Following this, the best aligned frame from each ORF is kept and marked for output. Lastly, during the UniProt reporting phase, all results are aggregated via sorting and grouping, and then submitted to UniProt to retrieve additional information corresponding to each read. This is saved in a tab delimited report. All three of these steps allow for output corresponding to work performed during that portion of the pipeline process.

Similar to the indexing process, the alignment process is also based heavily on the underlying BWA code, with modifications made to align in the amino acid space, including logging and output changed to reflect these IUPAC characters. This step in the pipeline also processes the ID and frame of each sequence, coordinating the reading frame filtering process documented above.
